# Personal attributes and attitudes to substance use disorder: A study among Jordanian undergraduate medical majors students

**DOI:** 10.1371/journal.pone.0263442

**Published:** 2022-02-22

**Authors:** Sawsan Abuhammad, Reem Hatamleh, Mohammad Alrawashdeh, Nasr Alrabadi, Tareq Mukattash, Mai Abuhammad, Kimberly Howard

**Affiliations:** 1 Maternal and Child Health Department, Faculty of Nursing, Jordan University of Science and Technology, Irbid, Jordan; 2 Department of Pharmacy, Faculty of Medicine, Jordan University of Science and Technology, Irbid, Jordan; 3 Department of Clinical Pharmacy, Faculty of Pharmacy, Jordan University of Science and Technology, Irbid, Jordan; 4 Health Center, Department of Emergency, Jordan University of Science and Technology, Irbid, Jordan; 5 Independent Researcher, Irbid, Jordan; GITAM College of Pharmacy: GITAM Institute of Pharmacy, INDIA

## Abstract

**Background:**

Emerging health professionals in undergraduate programs should be equipped to provide care to people with substance use disorder (SUD). The students’ personal attributes may impact their attitude toward those with SUD. This study aims to evaluate the impact of personal attributes of Jordanian undergraduate health students on their attitudes toward SUD and examine the relationship between the personal attributes and their devaluation and discriminatory (stigmatory) behaviour toward those with SUD.

**Method:**

A cross-sectional descriptive design was used to examine the attitudes and stigmatory behaviours. The data were collected between May to October 2017 with a structured questionnaire that consisted of three parts: 1) a data sheet to collect the socio-demographic characteristics of the participants, 2) the Acute Mental Health Scale (ATAMHS), and 3) the Devaluation-Discrimination Scale (DDS).

**Findings:**

Younger and females demonstrated a positive attitude toward those with SUD compared to older or male students. Age, gender, and previous experience with SUD are significant factors that affect their attitude.

**Conclusion:**

Identifying the attitude to people with SUD and personal attributes of emerging health professionals in Jordan will help identify the need to educate them prior to their entry into practice.

## Introduction

Illicit substance use is a global phenomenon and a public health burden. This condition also called substance use disorder (SUD) is a treatable condition. However, many of those who use the substance forgo treatment, leaving investigators puzzled about the nexus between healthcare and this population [1]. The stigma associated with substance use and negative attitudes held by healthcare professionals (HCPs) impact access to healthcare because these individuals avoid seeking help [2]. Therefore, it is vital to investigate the existence of stigmatizing attitudes toward patients with SUD to understand both the relationship between their beliefs and patient care. Such knowledge could guide HCPs to develop helpful interventions.

Research indicates that there is a relationship between the attitude of HCPs and the delivery of care. Nursing students’ characteristics, knowledge, and attitude about mental illness could predict the care provided to those with mental illness [3]. It was found that when HCPs have negative attitudes toward those who abuse substances, this can result in problems such as inaccurate medical diagnoses, including dismissal of symptoms as solely instigated by the patient’s illicit substance use [2]. Healthcare professionals can also see illicit substance use as largely self-inflicted, further complicating the attitudes about caring for these patients with poor compassion and low regard toward them [4]. In addition, illicit substance use is associated with criminality, as in many countries the use of illegal narcotics can result in criminal arrest, and additional legal consequences [5]. This also can lead to stigma, lack of empathy and low-quality care. Nevertheless, practicing HCPs are expected to care for people with any ailment without bias [4].

Personal characteristics can impact one’s attitude toward those with SUD. Few studies have shown that gender, education, income, knowing a drug abuser, and previous personal experience with substance users are factors that influence the HCP’s attitudes and discriminatory behaviors toward those who abuse substances [6–9]. These studies examined factors that affect the attitudes of practicing HCPs toward those who abuse substances. Only few studies examined the attitude of emerging HCPs to those with SUD, and that too not in the recent years [8, 10]. Data from the U.S. reveal that the number of people with SUD is on the rise [11]. As SUD is an emerging global problem, it is essential that future HCPs must be well equipped to manage this. However, there is a gap in the recent literature, particularly in the Jordanian context. Hence, an assessment of the attitudes of undergraduate students pursuing healthcare professions toward those with SUD would serve as a very important contribution to the literature. Gaining an understanding of their current attitudes and their personal attributes could help establish guidelines for recruiting the right personnel for providing care for these with SUD. In addition, the next generation of HCPs can be educated on this important topic. If a significant gap in the curriculum exists that could be remedied to equip them for practice. Therefore, this study aimed to evaluate (a) the impact of personal attributes of undergraduate health profession students on their attitudes toward those with SUD, and (b) examine the relationship between the personal attributes and their devaluation and discriminatory (stigmatory) behaviour toward those with SUD.

## Methods

### Study design

A cross-sectional descriptive design was used to study the attitudes of undergraduate students enrolled in health majors in a university in northern Jordan. A survey was used to collect data from 251 students. Institutional Review Board (IRB) of the University approved the study.

### Sample and setting

The participants were recruited from all health majors of the university in northern Jordan. Inclusion criteria were: age 18 and above, enrolled in in a health profession program (anywhere from first to the sixth year of study), and must read and write the Arabic language. Potential participants were excluded if they currently had SUD, or not registered in a health profession program. The sample size was calculated by using G-Power statistical analysis technology software (G-Power 3.1.9.2, 2014) through the use of the *F* statistic and a linear multiple regression model that included six predictor variables (gender, age, discipline, income, knowing an Illicit substance user and experience of caring for substance users) with a medium effect size, 0.8 power, and a significance of 0.05. The results showed that at least 200 participants were needed for the study to have statistical significance.

### Measures

The data were collected between May to October 2017 with a structured questionnaire that consisted of three parts: 1) a data sheet to collect the socio-demographic characteristics of the participants, 2) the Acute Mental Health Scale (ATAMHS), and 3) the Devaluation-Discrimination Scale (DDS). The ATAMHS and DDS were originally in English and permission to translate both to the Arabic language and use them were obtained. The original tools were translated into Arabic after getting permission from the original authors of these tools [12, 13], revere translated and verified for accuracy and consistency by two professors fluent in English and Arabic.

#### Socio-demographic characteristics

Demographic data consisted of gender, age, discipline, family income, and personal and professional experiences with illicit substance users. The researcher developed this tool.

#### Attitudes toward Acute Mental Health Scale (ATAMHS)

The ATAMHS is used to measure attitudinal data from the participants to increase knowledge on and around the subject of mental healthcare attitudes among health professionals [12]. The ATAMHS is a validated questionnaire that has been shown to have good internal reliability and a Cronbach’s alpha score of 0.72 [12]. This tool was used because illicit substance use is an addictive behavior that is considered a form of mental illness [12]. The ATAMHS scale covers five categories: 1) semantic differential, 2) care or control, 3) positive attitudes, illicit substance abuse 4) hard to help, and 5) therapeutic perspectives. The ATAMHS scale contains a total of 25 item questions/statements. The items are on a five-point Likert scale and measure negative-to-positive variance and the responses range from strongly disagree (1) to strongly agree (5).

#### Devaluation-Discrimination Scale

The Modified Devaluation-Discrimination Scale (DDS) measures the degree to which mental health patients experience devaluation and discrimination [13]. This tool was used to calculate the perceived amount of discrimination from undergraduate health profession students toward illicit substance users. The DDS consists of 12 items, which are scored using a five-point Likert scale with responses ranging from not at all (1) to a great deal (5) and includes six items that are reverse scored. The internal consistency of the scale was found to be .76 and reliability ranged from .72 to .88 (e.g., [13]).

### Ethical considerations

The researchers obtained prior approval from the IRB of Jordan University of Science and Technology to conduct this study (IRB no. 337–2017). Consent was informed and written consent was signed from the participants. The participants were told that participation was voluntary; Anonymity and confidentiality were assured to the participants. Data were stored in a locked cabinet in the principal investigator’s office.

### Recruitment and procedure

Upon obtaining IRB approval, the researchers developed promotional materials to facilitate recruitment. Leaflets with information on the purpose of the study and how to participate in the study were distributed to members of the colleges of health professions at the University via electronic mail. The e-mail included a description of the research being conducted; a request for permission to present the research study to the students, and information on how undergraduate students enrolled in health-related programs could participate in the study. After receiving feedback, the researchers visited the classes of the faculty members who allowed the research team to present the study information to the students. All the eligible participants were given a consent form that described the purpose of the study and they were asked to sign it before completing the questionnaire. The research group answered all the questions posed by the study participants prior to the completion of the questionnaire. The participants completed the survey on site and those who chose to complete it later dropped off the surveys at a designated location. It was estimated that the questionnaire would take 30–45 minutes to complete.

### Data analysis

Descriptive statistics were used to summarize the continuous and discrete variables by mean and standard deviation and frequencies and proportions, respectively. Two multiple regressions were applied: first to determine the factors that had an impact on devaluation and discrimination toward those with SUD and the second to determine the factors that affected their attitude. The assumption of the homogeneity of variance was tested and found met based on Levene’s statistics. All the tests were two-sided at a significance level of 0.05 and performed using the Statistical Package for the Social Sciences (SPSS version 25).

## Results

The final sample consisted of 251 students aged 21.3 ± 2.7 years. Approximately two thirds of the sample were female (67.3%), the majority were single (89.9%) and did not know a person with SUD (69.3%) or cared for a person with SUD (78.9%). The mean score for attitude toward SUD was 69.7 ± 11.7. The mean score for devaluation was 36.7 ± 5.1. The baseline characteristics of the total sample and by gender are presented in Table 1. Students from health professions were participated in the study.

**Table 1:** Baseline characteristics for the sample based on gender; mean ± SD or frequency (percentage).

Variable	Total (n = 251)	Male (n = 82)	Female (n = 169)	P-value
**Age**	21.3 ± 2.7	22.1 ± 2.2	21.0 ± 2.8	.001
**Marital Status**				
*Single*	225 (89.6)	69 (84.1)	156 (92.3)	
*Married*	12 (4.8)	4 (4.9)	8 (4.7)	
*Divorced*	14 (5.6)	9 (11.0)	5 (3.0)	
**Income**				.29
*≤ 3600 JD*	116 (46.2)	34 (41.5)	82 (48.5)	
*>3600 JD*	135 (53.8)	48 (58.5)	87 (51.5)	
**Education**				.001
*≤ 3rd year*	131 (52.2)	31 (37.8)	100 (59.2)	
*≥ 4th year*	120 (47.8)	51 (62.2)	69 (40.8)	
**Specialty**				< .001
*Nursing/Midwifery*	94 (37.5)	19 (23.2)	75 (44.4)	
*Medicine/Dentistry*	79 (31.5)	40 (48.8)	39 (23.1)	
*Pharmacy/Pharmacy D*	30 (12)	14 (17.1)	16 (9.5)	
*Other*	48 (19.1)	9 (11.0)	39 (23.1)	
**Father Education**				.04
*Never*, *Rarely*, *Sometime*	131 (52.2)	35 (42.7)	96 (56.8)	
*Usually*, *Always*	120 (47.8)	47 (57.3)	73 (43.2)	
**Mother Education**				< .001
*Never*, *Rarely*, *Sometime*	157 (62.5)	38 (46.3)	119 (70.4)	
*Usually*, *Always*	94 (37.5)	44 (53.7)	50 (29.6)	
**Know person with addiction**				< .001
*Yes*	77 (30.7)	38 (46.3)	39 (23.1)	
*No*	174 (69.3)	44 (53.7)	130 (76.9)	
**Experience caring for addicted person**				< .001
*Yes*	53 (21.1)	21 (25.6)	19 (11.2)	
*No*	198 (78.9)	61 (74.4)	150 (88.8)	
**Attitude toward drugs, Total Score**	69.7 ± 11.7	66.6 ± 14.4	71.2 ± 9.8	.01
**Devaluation, Total Score**	36.7 ± 5.1	36.9 ± 4.9	36.6 ± 5.3	.71

There were significant differences in the baseline characteristics of the male and female participants. Multiple regression analysis was used to develop a model for predicting the factors that affected the attitudes of the participants toward illicit substance users. In this first predictor model, each of the predictor variables had a significant (p < .01) correlation with attitudes and with perceived attitude toward illicit substance users, but only age, gender, and previous experience caring for a person with SUD had a significant partial effect in the full model (p < .05) as shown in Table 2.

**Table 2:** Multivariate regression analysis for the factors affecting attitude towards illicit drug users.

Variable	*Coefficient*	*Confidence Interval*	*P-value*
**Age**	-0.650	(-1.26, -0.04)	**0.038**
**Gender, Female**	3.203	(-0.02, 6.43)	0.052
**Marital Status**	2.26	(-0.74, 5.26)	0.139
**Income**	-0.059	(-2.94, 2.82)	0.968
**Education**	0.074	(-3.35, 3.49)	0.966
**Specialty**	-0.367	(-1.74, 1.01)	0.600
**Father Education**	0.174	(-3.18, 3.53)	0.919
**Mother Education**	0.405	(-3.11, 3.92)	0.821
**Know illicit drug user**	-1.596	(-4.93, 1.73)	0.346
**Experience caring for illicit drug user**	-5.292	(-9.49, -1.10)	**0.014**

The second predictor model was able to describe the variance in the devaluation- discrimination behavior of the participants toward those with SUD. The results of the multivariate regression analysis of the factors that affected devaluation behavior toward illicit substance users are displayed in Table 3. Knowing a person with SUD was the only significant predictor of a positive attitude toward (B = 2.303, p = .003) those with SUD. Education and other factors did not have any significant effect.

**Table 3:** Multivariate regression analysis for the factors affecting for devaluation behavior towards illicit drug users.

Variable	*Coefficient*	*Confidence Interval*	*P-value*
**Age**	0.122	(-0.15, 0.40)	0.382
**Gender**	0.506	-0.94, 1.95)	0.490
**Marital Status**	-0.757	(-2.10, 0.58)	0.267
**Income**	-0.6	(-1.89, 0.69)	0.360
**Education**	1.021	(-0.51, 2.55)	0.189
**Specialty**	-0.301	(-0.92, 0.31)	0.336
**Father Education**	-0.138	(-1.64, 1.36)	0.856
**Mother Education**	-0.2	(-1.77, 1.37)	0.803
**Know illicit drug user**	2.303	(0.82, 3.79)	**0.003**
**Experience caring for illicit drug user**	0.833	(-1.04, 2.71)	0.383

## Discussion

This research study examined the attitudes and stigmatory behavior of students in undergraduate health professions toward those with SUD in Jordan. Descriptive statistics were used to understand the impact of the target population’s demographic data on their attitudinal data. The results indicated generally positive attitudes toward those with SUD among the sample population; however, some significant variations in attitudes and discrimination were correlated to gender and educational level. In this study, therapeutic attitudes were defined as a hypothetical construct of an individual’s level of like or dislike of a given variable within the healthcare setting; a positive attitude was considered as having compassion for those with SUD, while a negative attitude was associated with devaluation of this group [4]. It is important to evaluate attitudinal and discrimination scale data because they can lead to expression in the healthcare environment which could impact the quality of care [14]. In addition, the data can also be used to inform interventions and thereby help to prevent the impact of partialities on healthcare services [14].

The questionnaire was used to explore the attitudes and discrimination and their relationship to the demographic data are given in Tables 2 and 3. As regards the demographic data, there was a significant correlation with gender. Specifically, female undergraduate health profession students were less negative and discriminatory than their male counterparts. This supports the finding of previous studies where female undergraduate healthcare students were found to be more positive toward those with SUD compared to their male peers [15, 16]. However, it should be noted that the authors of both of those studies suggested that additional variables may have influenced the correlation, and this suggestion could also be relevant to this study. No recent studies that examined these were found in the literature.

Regarding the effect of educational level on their attitude to those with SUD, the results showed that students in their junior year were more negative than those in their senior years. This is consistent with the findings from a previous study [17]. Again, it should be noted that, while the educational level was found to be significant, it could also share a relationship with additional variables such as the amount of professional contact and even non-professional exposure to illicit substance users. Knowledge deficit could be one of the reasons and possible the senior students probably learned about SUD. Knowledge deficit was identified as a major problem of attitudinal differences among practicing professional [11].

As for contact and experience with those with SUD, there was a statistically relevant correlation between these two variables and both attitude and discriminatory behavior. Participants who had more contact and experience with SUD were more negative and discriminatory toward the users when compared to their peers with less experience. In contrast, a previous study involving practicing professionals showed that in-service training sessions related to SUD helped to increase positive attitudes among staff [6]. Schools should consider adding content addressing SUD in the curriculum so that graduates will be prepared to meet the need of the community on entry to practice. Regular in-services should be provided by employers to update the existing professional to meet the dynamic needs of the population.

Unlike the findings in previous studies, this study did not find any correlations with more negative or more positive results based on their discipline. Earlier studies examined the practitioners’ experience with SUD in the healthcare environment and the type of contact (i.e., whether the person is in recovery or relapse], which influenced their attitude [2]. However, as these were practicing professionals with varying ages and experiences; hence this cannot be compared to our study. Therefore, further research among emerging Medical Majors students could help to determine whether age, gender, educational level, contact, experience, as well as other measurable variables influence their attitude.

### Implications and recommendation for future

This study documented mixed responses from participants according to the demographic data, while also indicating that there was general positivity toward those with SUD, particularly among the younger students, a finding that contrasts with the literature that there is negativity among healthcare professionals working with those suffering from SUD [2]. This suggests that the institutions responsible for educating HCPs should be seeking to help further shift therapeutic attitudes among students by including appropriate evidence-based materials in their curriculum that will better prepare them to work with this group pf people in a way that successfully reduces biases [2, 3]. It worth to mention that the curriculums in Jordan in general do not include any information about the discrimination and the impact of the discrimination on the addicted patients and how prevent them in having appropriate health care. Moreover, using an attitudinal scale to assess discriminatory attitudes before and after course completion, and obtaining patient feedback help provide data-driven measures of success. It is also important to consider the significance of parents’ education, marital status, and how medical educators can use these in teaching and curriculum planning for the students and their parents.

Furthermore, SUD should be embedded into continuing education/continuing professional development programs, which will raise awareness among practicing HCPs and reduce biases, thus systematically reverse the negativity documented in the literature [18, 19]. Also, multidimensional and interdisciplinary approaches that consider, for instance, public health strategies, could also lead to the development and provision of more effective tools to address the issue of substance abuse [1].

Overall, additional interventions are needed to help prepare HCPs to avoid discriminating against patients, provide better medical care and achieve more sustainable rehabilitation results. Such efforts could help prevent the causal influence of persistent negativity and pessimism and should continue even if future research documents an increase in positive attitudes and behaviors among students and/or practitioners.

### Limitations

This study was conducted in ne university in Jordan using a small sample. The measuring tools were translated into Arabic and some clarity may be lacking. Even in Jordan, the Arabic language may have minor variations and some students might have interpreted some words differently. Perceived social acceptability might have prevented some students from stating their real feelings. The students were at varying levels of education which might have given them different exposure to information about SUD. Having relatives or knowing people who suffered from SUD and the close-knit family structure of Jordan may prevent students from expressing the true feelings. That makes these findings less generalizable. Large scale studies involving several universities in Jordan may provide a better picture of the attitudes and discriminatory behaviour patterns of undergraduate health students.

## Conclusion

This study is novel since it is the first study in Jordan to discusses attitude toward addicted patients. Healthcare professionals play a very significant role in providing the right medical attention to patients from all walks of life. Quality effective healthcare delivery can have a tremendous impact on health outcomes. Therefore, it is crucial to find areas where clear deficiencies exist so that optimal care can be facilitated. The research data shows that there is a significant need to improve healthcare services for those with SUD. Also, when the statistical data shows that those with SUD are not very likely to seek services, and when they seek services, they experience significant stigma, studies such as this will identify a need to prepare HCPs. This study showed that younger students in healthcare majors have a more positive attitude toward illicit substance users with some variations in the data that correlated with other demographic characteristics. While the results of this study might not be generalizable, additional research involving the younger healthcare students may help them develop the right attitude toward those with SUD. In addition, such research could involve other Middle Eastern countries and the finding s and interventions could impact healthcare education beyond Jordan. Thus, there should be profound motivation among all stakeholders to make modifications to healthcare education and practices to successfully address this public health issue.

## Supporting information

S1 Data (XLS)
